# Reliability of nasofibroscopy for the evaluation of adenoid hypertrophy and its correlation with clinical symptoms

**DOI:** 10.1016/j.bjorl.2023.101307

**Published:** 2023-08-25

**Authors:** Juliana Pascutti Sant’Ana, Isabella Cristina Fasanella Mastrandonakis, Renata Santos Bittencourt Silva, Andre de Campos Duprat, Caio Gomes Floriano, Marcel Menon Miyake

**Affiliations:** a Santa Casa de São Paulo, Departamento de Otorrinolaringologia, São Paulo, SP, Brazil; b Santa Casa de São Paulo, São Paulo, SP, Brazil

**Keywords:** Adenoid hipertroph, Child, Nasal endoscopy, Obstrutive sleep apnea, Quality of life

## Abstract

**Objective:**

To analyze the reliability of estimating the percentage of rhinopharyngeal obstruction by the adenoid using nasofibroscopy and its correlation with clinical symptoms.

**Methods:**

Cross-sectional observational study was conducted, involving 80 patients between 4 and 14 years old, recruited from Santa Casa of São Paulo general otorhinolaryngology outpatient service during the years of 2020 and 2021. All patients underwent nasal endoscopy examination, and the recorded videos were randomly assigned to four evaluators in two different sessions, with a minimum interval of 1 month. The evaluators estimated the percentage of rhinopharyngeal obstruction caused by the adenoid. Intra- and inter-evaluator correlations were established by comparing the reports from each evaluator. The data were compared to the Pro Image J Software report, that also estimates a percentage of obstruction by computer graphics. To correlate the grading of obstruction with clinical symptoms, all patients completed the OSA (Obstructive Sleep Apnea) 18 questionnaire, a validated tool for assessing sleep apnea in children. The questionnaire data were then compared to the average scores assigned by the evaluators.

**Results:**

Satisfactory intra- and inter-rater correlations were observed, and the results were consistent with the Pro Image J Software. However, no correlation was found between the percentage of obstruction and the severity of clinical symptoms.

**Conclusion:**

Nasofibroscopy demonstrates good reliability in assessing a percentage of rhinopharyngeal obstruction caused by adenoids. However, there is no correlation between the degree of obstruction of the rhinopharynx and the clinical symptoms.

**Level of evidence:**

4.

## Highlights

• Nasofibroscopy is a reliable test for assessing adenoid hypertrophy in children.
• No correlation has been demonstrated between adenoid hypertrophy and quality of life.
• Body mass index as an independent factor in the impact on quality of life.

## Introduction

Adenoid hypertrophy is one of the most common alterations of the upper airways in childhood^1^ and can cause several repercussions on the child's quality of life and development. The narrowing of the airway in the rhinopharynx can restrict the passage of air during nasal breathing, resulting in obstruction, oral breathing, and snoring and, in more advanced cases, obstructive sleep apnea and alteration in orofacial development.^2^

Nasofibroscopy is considered the gold standard exam for assessing adenoid hypertrophy, enabling direct visualization of the rhinopharynx and adenoid lymphoid tissue.^3^ However, this method has some practical limitations. In clinical practice, most examinations rely on subjective estimation of rhinopharyngeal obstruction caused by the adenoid, given as a percentage by the examiner, which is a method that is still poorly established in the literature.^4^, ^5^ Therefore, it cannot be concluded that this method is reliable and reproducible, which can potentially impact clinical decision-making and direct management based on inaccurate information.

Unlike tonsillar hypertrophy,^6^, ^7^ the correlation between the degree of adenoid hypertrophy and the intensity of respiratory symptoms, such as nasal obstruction and nocturnal snoring, is still not well established.^8^, ^9^ One of the reasons is its frequent association with other factors, such as rhinitis. hypertrophy, turbinate hypertrophy, and tonsillar hypertrophy itself, making it difficult to study the isolated impact of the enlarged adenoid. In the case of a multifactorial condition, understanding the real impact of adenoid hypertrophy on respiratory symptoms and whether, in fact, the larger the adenoid, the greater the clinical repercussions, is of fundamental importance to guide the management of these patients.

The limitations of the technique, as well as the subjectivity in interpreting adenoid hypertrophy diagnosed by nasopharyngoscopy, can impact clinical reasoning and lead to management decisions based on imprecise information. We did not find studies correlating the degree of adenoid hypertrophy with the intensity of symptoms such as nasal obstruction or nocturnal snoring. Additionally, we found no studies that define a specific percentage of rhinopharyngeal obstruction caused by the adenoid that suggests the need for adenoidectomy. Several studies have evaluated the correlation between hypertrophy of the palatine tonsils and respiratory symptoms,^6^, ^7^ but the relationship between isolated adenoid hypertrophy and its clinical repercussions remains limited.^8^, ^9^

Thus, this study seeks to assess the reliability of estimating degree of rhinopharyngeal obstruction by the adenoid through nasofibroscopy and to correlate it with the intensity of nasal clinical symptoms, nocturnal snoring, and sleep apnea.

## Methods

Cross-sectional observational study that analyzes the reliability of adenoid evaluation by nasofibroscopy and correlates the degree of rhinopharyngeal obstruction with clinical symptoms.

The study was approved by the Ethics and Research Committee (CAAE 29266820.1.0000.5479). Data were stored on the RedCap platform.

The patients were screened at the various outpatient clinics of the Department of Otorhinolaryngology at Santa Casa of São Paulo over the years 2020 and 2021.

Patients of both sexes, between 4 and 14 years old, with nasosinusal complaints and/or nocturnal snoring, who would undergo nasofibroscopy for clinical investigation, were selected. Patients who presented craniofacial malformations, Down Syndrome, congenital diseases, neuromuscular diseases, immunodeficiencies, current symptoms of upper airway infection, obstructive nasal septum deviation, and patients with Grade III or IV tonsillar hypertrophy were excluded. Patients who did not tolerate the examination or who presented poor quality videos that made it impossible to visualize the adenoid were also excluded.

After being instructed about the risks and benefits of participating in the study and agreeing to participate, patients and family members signed the informed consent and assent and answered a clinical and demographic questionnaire (Annex 1). To determine the clinical outcome, they answered the OSA 18 (Obstructive Sleep Apnea) questionnaire (Annex 2),^10^ translated into Portuguese and validated for assessing sleep apnea in children, consisting of 18 items divided into 5 areas: sleep disorders, physical symptoms, emotional symptoms, functions of daily life and concern of caregivers. The sum of the items results in a total that can vary from 18 to 126, with values below 60 having a small impact on quality of life. From 60 to 80 moderate impact on quality of life. Values above 80 have an important impact on quality of life.^10^

All patients underwent nasofibroscopy, in a sitting position, with a flexible fiberoptic nasofibroscope (OlympusTM ENFP4, 3.5 mm), with 250-watt halogen light, under topical anesthesia in both nasal cavities (4% lidocaine gel). Parents were informed how the exam would be performed and decided whether the children would be sitted alone or on their laps. No other immobilization method was used, and the examination was interrupted at any sign of discomfort. All videos ensured that the choana region was filmed during inspiration, with the palatal musculature relaxed, the moment for better assessment of rhinopharyngeal obstruction by the adenoid. The exam was recorded and edited in order to preserve the patient's identification.

The group of evaluators was composed of four independent otorhinolaryngologists, with a minimum of five years of clinical experience, who are not study authors and did not have access to research protocol data. All were instructed to choose the moment for the best evaluation of the choana, soft palate relaxed during inspiration and the fiber being located in the distal position of the inferior turbinate. The video clips were sent to the evaluators, numbered by codes and without identification, in two different moments (T1 and T2) with an interval of at least one month between them. In the second evaluation, the order of the videos was randomized to avoid the influence of the first evaluation. The examiners did not have access to the grading attributed by themselves in the first evaluation, nor the grading attributed by the other evaluators. In each analysis, examiners were instructed to subjectively estimate a percentage of rhinopharyngeal obstruction by adenoid tissue.^8^

**Figure 1:**
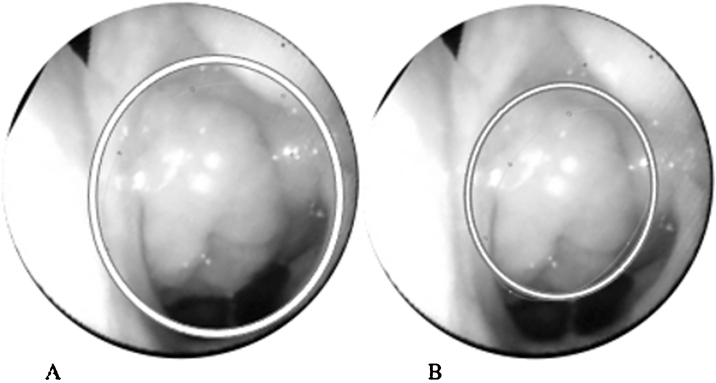
Nasal endoscopy to evaluate the rhinopharynx using the Pro Image J Software. (A) Area of the choana estimated by Software Pro Image J. (B) Area of adenoid tissue estimated by Software Pro Image J.

In addition to the subjective classifications, the videos were also digitally evaluated using the Pro Image J Software, which estimates the degree of rhinopharyngeal obstruction by the adenoid. In this software, a fifth evaluator delimited the area occupied by the adenoid and the choana area, as shown in Fig. 1. With these data, the software calculated the percentage of obstruction of the choanas by the adenoid tissue (Fig. 1).^11^

### Statistical analysis

The sample size was calculated based on Pearson's correlation, adopting a significance level of 5%, test power of 80%, and an initial correlation between obstruction of the rhinopharynx by adenoid and clinical symptoms of 0.4 was assumed, resulting in a minimum sample of 50 patients.

Intra-rater reliability (between T1 and T2) was established by Pearson's correlation, being considered high when *r* >0.7 and *p* < 0.05.

Once the high intra-rater reliability was accepted, the mean response of the patient of each rater was used for the inter-rater reliability study, through the Intraclass Correlation Coefficient (ICC). It was adopted as a reliable ICC greater than 0.7.^12^, ^13^

Pearson's correlation was used to study the relationship between: (1) Subjective measurement of rhinopharyngeal obstruction by the adenoid, and objective measurement, by Pro ImageJ Software; (2) Obstruction of the rhinopharynx by the adenoid, both subjectively and objectively, and the intensity of the clinical symptoms determined by the OSA-18.

To explore the relationship between potential risk factors that could influence correlation between rhinopharyngeal obstruction by the adenoid and the impact on quality of life (OSA-18), Pearson's correlation was used for qualitative risk factors, and Student's *t* for the quantitative ones. Risk factors that presented *p* < 0.20 were selected for analysis of covariance, with OSA-18 being the dependent variable. For all analyses, a significance level of 5% was adopted. All analyzes were performed in SPSS 25.0.

## Results

**Figure 2:**
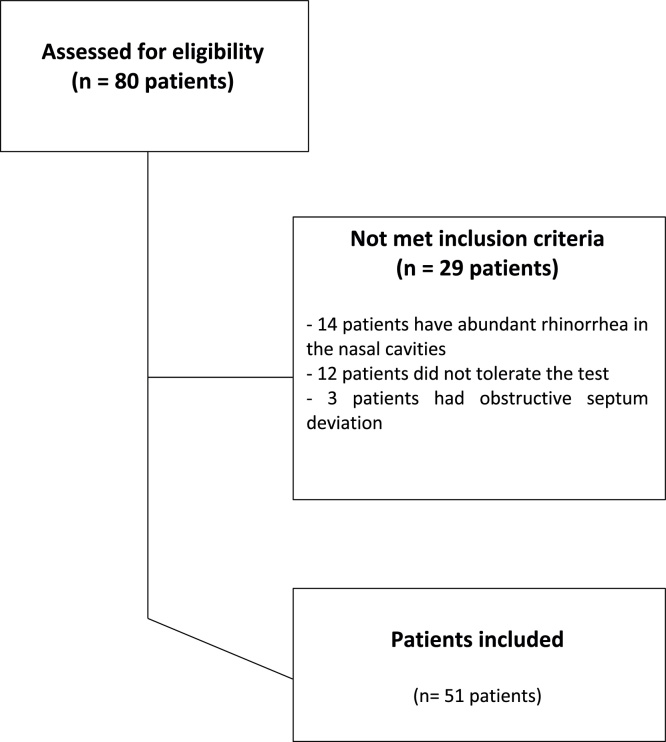
Flowchart representing the selection criteria applied to the study patients.

Eighty patients from the General Otorhinolaryngology outpatient clinic of XXX were screened. Among them, 29 were not included in the analysis (Fig. 2), as 12 patients did not tolerate the test because they felt uncomfortable during the procedure, 14 patients had abundant rhinorrhea in the nasal cavities and 3 patients had obstructive nasal septum deviation.

The demographic and clinical characteristics of the included patients are described in Table 1.

**Table 1:** Clinical and demographics characteristics.

	Frequency (%)
Sex	
Female	19 (37%)
Male	32 (63%)
Ethnicity	
White	6 (11%)
Brown	20 (39%)
Black	25 (49%)
Clinical symptoms	
Substitute mouth breathing	41 (80%)
Night snoring	34 (66%)
Nocturnal snoring and witnessed apneas	17 (33%)
Nasal allergic complaints	43 (84%)
Current nasal treatment	27 (52%)
Physical exam	
Retrognathia	5 (9%)
High palate	39 (76%)
Inferior turbinate hypertrophy	42 (82%)
OSA-18	
Hight impact	14 (27%)
Moderate impact	15 (29%)
Low impact	22 (43%)
Mean (SD)	
Age	7.7 years (2.8)
BMI	19.62 (5,8)
Adenoid size	59.8 (19.7)

**Figure 3:**
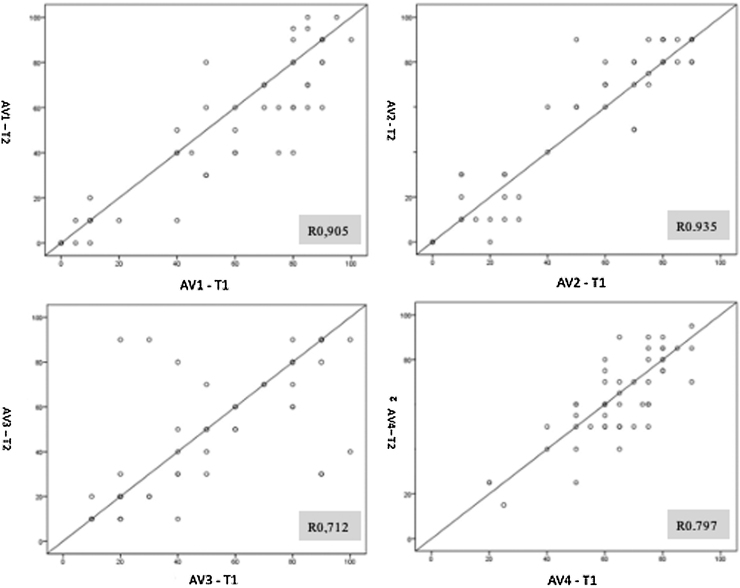
Scatter diagrams representing intra-rater correlation at T1 and T2.

The intra-rater correlation coefficient was calculated based on T1 and T2 scores with Pearson's ratio. The 4 evaluators showed good individual correlation *r* >0.7 and *p* < 0.001: Evaluator 1 (AV1) *r* = 0.905; Evaluator 2 (AV2) *r* = 0.935; Evaluator 3 (AV3) *r* = 0.702; Evaluator 4 (AV4) *r* = 0.797 (Fig. 3).

Inter-rater reliability was calculated using the Intraclass Correlation Coefficient (ICC) with an ICC result of 0.768 (95% IC 0.725‒0.875).

**Figure 4:**
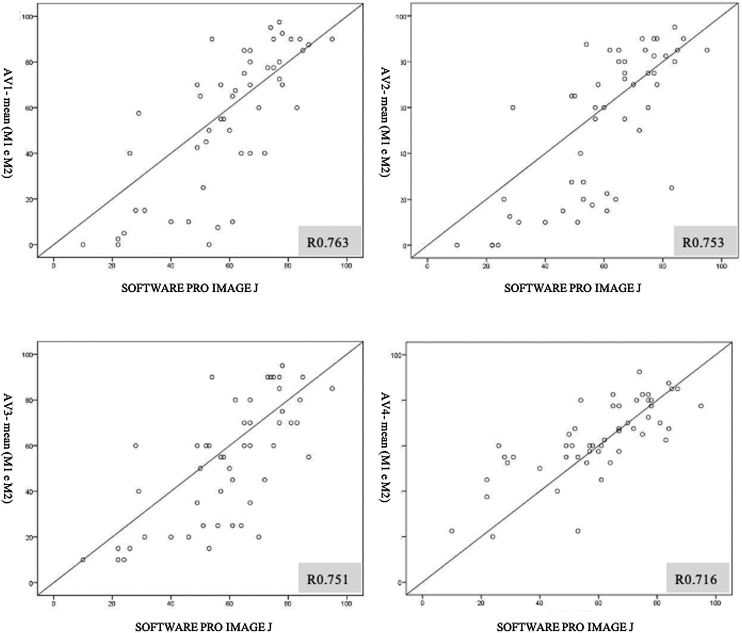
Scatter diagram comparing the estimated percentage of obstruction of the rhinopharynx by the adenoid, established by the average of the evaluators at T1 and T2, compared to the estimated percentage of obstruction established by the Software Pro Image J (Objective).

For each evaluator, the average of the results between T1 and T2 was calculated and compared to the findings of the Software Pro Image J, through Pearson's correlation. All showed correlation *r* > 0.7 and *p* < 0.001: Evaluator 1 (AV1) *r* = 0.763; Evaluator 2 (AV2) *r* = 0.753; Evaluator 3 (AV3) *r* = 0.716; Evaluator 4 (AV4) *r* = 0.751. (Fig. 4)

**Figure 5:**
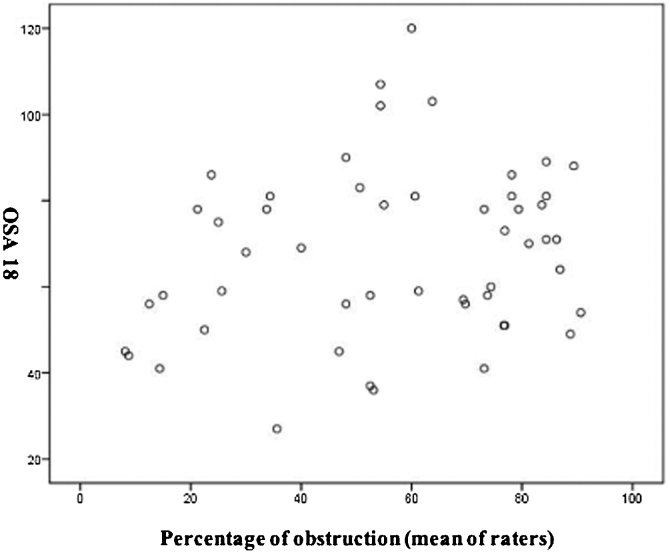
Correlated to the average of the results of adenoid hypertrophy between T1 and T2 of the four evaluators using Pearson's correlation.

Of the 51 participants, 22 patients had a low impact on the quality of life assessed by the OSA-18, 15 had a moderate impact and 14 had a high impact. The final OSA-18 score was correlated to the average of the results of adenoid hypertrophy between T1 and T2 of the four evaluators using Pearson's correlation (Fig. 5). There was no correlation between degree of obstruction and intensity of clinical symptoms (OSA-18) (*r* = 0.215; *p* = 0.13).

**Figure 6:**
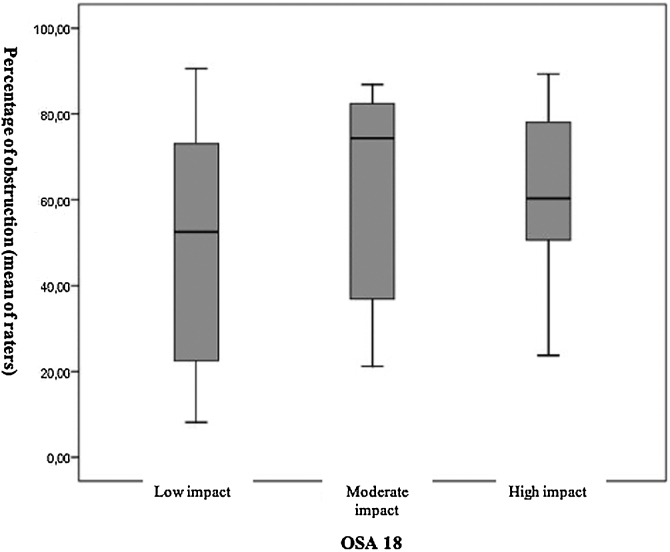
Distribution of the degree of obstruction of the rhinopharynx, given by the average of the evaluators, with the three categories of quality of life, determined by the OSA-18 questionnaire.

Moreovere, when evaluated the degree of obstruction of the rhynopharynx by the OSA-18 categories, no significant difference was observed between the three categories (*p* = 0.130) (Fig. 6).

Allergic rhinitis, inferior turbinate hypertrophy, age, current nasal treatment, and BMI were associated with OSA-18, with *p* < 0.20, and were selected for analysis of covariance. In this analysis, only the BMI factor was significant (*p* = 0.004). Thus, the higher the BMI, the greater the OSA-18 score. No correlation was observed between adenoid size and OSA-18 regardless of BMI (*p* = 0.284).

## Discussion

Although nasofibroscopy is considered the gold standard exam in the diagnosis of adenoid hypertrophy, most studies use classifications that are not currently used in clinical practice.^9^, ^14^ The use of the percentage of rhinopharyngeal obstruction by the adenoid tissue is frequently used in otorhinolaryngological offices, but there are few studies that evaluate its validity. This study observed that this method is reliable and reproducible when assessed by the same examiner in two different moments, by different examiners and also when compared to Software Pro Image J.

In the study, all four evaluators demonstrated a good correlation between T1 and T2 times (R > 0.7). However, although it is a good correlation, it is not excellent (R > 0.9). This could be improved if the evaluators were instructed to use anatomical parameters, for example, to determine the degree of obstruction by the adenoid tissue. We purposely refrained from providing such instructions in order to maintain the dynamics of clinical practice in the office.

Another observation pertains to the comparison between the Pro Image J software and the evaluators (objective vs. subjective). All showed a good correlation (R > 0.7), but this correlation varied between 0.93 and 0.7. Due to the limited number of examiners (only four), it was not possible to determine the reasons for this variance.

Once the method's reliability was confirmed, we assessed the correlation between the percentage of obstruction and the severity of clinical symptoms using the OSA-18 questionnaire. No correlation was observed, meaning that a more obstructed adenoid did not necessarily result in a more compromised quality of life. Evaluating the impact of the adenoid on these symptoms in isolation is challenging due to the multifactorial nature of sleep apnea, where several other factors may be involved. To ensure sample homogeneity and enhance the internal validity of the study, we applied strict inclusion criteria, excluding significant factors that could interfere with the impact of adenoids, such as tonsil hypertrophy and rhinorrhea/upper airway infection. Furthermore, we performed a linear model analysis of covariance to account for additional factors that might have influenced the results, including BMI, allergic rhinitis, current nasal treatment, and hypertrophic turbinates. Among these factors, only BMI exhibited a significant difference, indicating that higher BMI scores were associated with higher OSA-18 scores.

On the other hand, the strict inclusion criteria resulted in the exclusion of 14 patients who experienced rhinorrhea during the examination. Additionally, 12 patients were unable to tolerate the procedure, as it is not our service's protocol to use any patient restraint methods. For future studies, we intend to conduct pre-tests to evaluate patients' tolerance to nasal manipulation, ensuring their inclusion.

One of the main limitations of this study is the lack of objective examination to compare with the results evaluated by the examiners. However, the Pro Image J software, despite not being an objective method (it depends on a researcher to delimit the structures), is the best comparison parameter we had available since it is based on the gold standard method (nasofibroscopy) and avoids the limitations of imaging exams. Radiography and computed tomography of the cavum can show great variability in the assessment of adenoid size according to the incidence of the examination or whether the palate muscles are contracted at the time of examination, in addition to exposure to radiation and cost.^15^, ^16^, ^17^ Therefore, we did not perform imaging tests in this study.

This study has the potential to impact the clinical practice of otorhinolaryngologists and optimize diagnosis, interpretation and, mainly, procedures. The presented results confirm that the classification of adenoid hypertrophy through a percentage estimate is a reliable, reproducible and consistent method, supporting its use in clinical practice. However, the absence of correlation between the intensity of rhinopharyngeal obstruction by the adenoid and the clinical symptoms evaluated by the OSA-18 call an alert for the way of interpreting this exam. The nasofibroscopic evaluation of adenoid hypertrophy is a complementary exam and, therefore, must complement a reasoning based on the clinical history and the physical exam in order to arrive at an adequate conduct. According to the results of this study, it is not possible, for example, to conclude that adenoid hypertrophy above a certain percentage, regardless of clinical symptoms, is indicative of the need for more invasive therapies, such as adenoidectomy. Thus, adenoidectomy should be indicated based on persistent respiratory symptoms refractory to clinical treatment, corroborated by the nasofibroscopic finding of adenoid hypertrophy.^18^, ^19^

Cassano et al. evaluated 7621 children with adenoid hypertrophy and observed that among those with Grade IV (100% obstruction of the rhinopharynx), there was a strong correlation with clinical repercussions such as sleep apnea and recurrent otitis. Among in 881 patients (54.7%) belonging to the Grade III group (>75% obstruction of the rhinopharynx), medical treatment was effective in controlling nasal obstruction and related pathologies and complications; and therefore, did not require surgical intervention. This finding supports the observations of our study and the approach of our team, which recommends more invasive therapeutic alternatives for patients with more severe clinical involvement, rather than relying solely on the presence of hypertrophic adenoids on examination. It is important to note that Cassano et al.'s study included patients with other conditions that can impact sleep quality, such as tonsil hypertrophy and nasal secretions.^18^

Finally, we reinforce the importance of anamnesis and physical examination in the evaluation of respiratory complaints in children and we understand that nasofibroscopy is a reliable complementary exam, but that by itself it is not capable of determining the intensity of symptoms or prognosis.

## Conclusion

The findings of this study allow us to conclude that the evaluation of the percentage of rhinopharyngeal obstruction by the adenoid, through nasofibroscopy, is a reliable, consistent, and reproducible method for the evaluation and classification of adenoid hypertrophy. However, no correlation was observed between the degree of rhinopharyngeal obstruction by the adenoid and the intensity of clinical symptoms.

## Conflicts of interest

The authors declare no conflicts of interest.
