# Supplementary material for: Reliability of nasofibroscopy for the evaluation of adenoid hypertrophy and its correlation with clinical symptoms
Source: Braz J Otorhinolaryngol. 2023 Aug 25;89(5):101307. doi: 10.1016/j.bjorl.2023.101307 (PMC10493505; doi:10.1016/j.bjorl.2023.101307)
Supplement: Supplementary file 1 [file mmc1.pdf]

Anexo I: Questionário Demográfico e Clínico

Registro no Estudo:

Data de inclusão (DD/MM/ANO):

<u>Informações do paciente:</u>

Nome: Registro Santa Casa (MV/AT):

Nome do responsável: Contatos:

<u>Dados Clínicos:</u>

Idade: ___________ **Sexo**: ☐masculino ☐feminino

Etnia: ☐negro ☐branco ☐pardo ☐amarelo ☐outros:_______

IMC: ☐<25 ☐25-30 ☐30-35 ☐>35

Respiração oral: ☐sempre ☐maioria das vezes ☐às vezes

Apresenta queixa de roncos e apneia? ☐Não ☐Apenas roncos ☐Roncos e apneia

Queixas alérgicas nasais crônicas? ☐Não ☐Sim

Está em tratamento nasal atualmente? ☐Não ☐Sim

Asma? ☐Não ☐Sim

Tabagismo passivo? ☐Não ☐Sim

Exame físico

Retrognatia: ☐Não ☐Sim

Oroscopia:

- Amigdalas grau: ☐0 ☐1 ☐2

- **Palato Ogival:** ☐sim ☐não

Rinoscopia:

- **Cornetos hipertróficos:** ☐Não ☐Sim

- **Cornetos pálidos:** ☐Não ☐Sim

- **Rinorréria:** ☐Não ☐Sim ☐Mucopurulenta
