# Supplementary material for: Reliability of nasofibroscopy for the evaluation of adenoid hypertrophy and its correlation with clinical symptoms
Source: Braz J Otorhinolaryngol. 2023 Aug 25;89(5):101307. doi: 10.1016/j.bjorl.2023.101307 (PMC10493505; doi:10.1016/j.bjorl.2023.101307)
Supplement: Supplementary file 2 [file mmc2.pdf]

Anexo II

Questionário OSA 18 versão língua portuguesa.

OSA-18 Versão Portuguesa (OSA-18-pv) Data: ___/_____/___							
Nome:_______________________							
Em cada uma das questões seguintes, faça por favor um círculo à volta do número que melhor descreve a frequência de cada sintoma ou problema nas últimas 4 semanas. Assinala apenas um número por questão. Obrigado.							
	Nunca	Quase Nunca	Poucas Vezes	Algumas Vezes	Bastantes vezes	Quase Sempre	Sempre
Distúrbio do Sono							
Nas últimas 4 semanas, com que frequência o seu filho teve...							
... ressonar alto?	1	2	3	4	5	6	7
... paragens na respiração durante a noite?	1	2	3	4	5	6	7
... engasgos ou respiração ofegante enquanto dormia?	1	2	3	4	5	6	7
... sono agitado ou despertares frequentes do sono?	1	2	3	4	5	6	7
Sintomas Físicos							
Nas últimas 4 semanas, com que frequência o seu filho teve...							
... respiração bucal por obstrução nasal?	1	2	3	4	5	6	7
... resfriados ou infecções das vias aéreas superiores?	1	2	3	4	5	6	7
... secreção e congestão nasal?	1	2	3	4	5	6	7
... dificuldade em engolir alimentos?	1	2	3	4	5	6	7
Problemas emocionais							
Nas últimas 4 semanas, com que frequência o seu filho teve...							
... alterações do humor ou acessos de raiva?	1	2	3	4	5	6	7
... comportamento agressivo ou hiperactivo?	1	2	3	4	5	6	7
... problemas disciplinares?	1	2	3	4	5	6	7
Problemas do quotidiano							
Nas últimas 4 semanas, com que frequência o seu filho teve...							
... sonolência diurna excessiva?	1	2	3	4	5	6	7
... episódios de falta de atenção ou concentração?	1	2	3	4	5	6	7
... dificuldade ao levantar da cama de manhã?	1	2	3	4	5	6	7
Opinião do Informante							
Nas últimas 4 semanas, com que frequência os problemas acima descritos...							
... causaram preocupação com a sua saúde?	1	2	3	4	5	6	7
... preocuparam-no pelo seu filho não poder respirar ar suficiente?	1	2	3	4	5	6	7
... interferiram com as suas actividades diárias?	1	2	3	4	5	6	7
... deixaram-no frustrado?	1	2	3	4	5	6	7
